# An Integration of GPS with INS Sensors for Precise Long-Baseline Kinematic Positioning

**DOI:** 10.3390/s101009424

**Published:** 2010-10-20

**Authors:** Hungkyu Lee

**Affiliations:** Department of Civil Engineering, Changwon National University, 9 Sarim-dong, Changwon, Gyeongnam, 641-773, Korea; E-Mail: hkyulee@changwon.ac.kr; Tel.: +82-55-213-3777; Fax: +82-55-285-9491

**Keywords:** navigation sensor integration, Global Positioning System (GPS), Inertial Navigation System (INS), multiple reference stations

## Abstract

Integrating the precise GPS carrier phases and INS sensor technologies is a methodology that has been applied indispensably in those application fields requiring accurate and reliable position, velocity, and attitude information. However, conventional integration approaches with a single GPS reference station may not fulfil the demanding performance requirements, especially in the position component, when the baseline length between the reference station and mobile user’s GPS receiver is greater than a few tens of kilometres. This is because their positioning performance is primarily dependent on the common mode of errors of GPS measurements. To address this constraint, a novel GPS/INS integration scheme using multiple GPS reference stations is proposed here that can improve its positioning accuracy by modelling the baseline-dependent errors. In this paper, the technical issues concerned with implementing the proposed scheme are described, including the GPS network correction modelling and integrated GPS/INS filtering. In addition, the results from the processing of the simulated measurements are presented to characterise the system performance. As a result, it has been established that the integration of GPS/INS with multiple reference stations would make it possible to ensure centimetre-level positioning accuracy, even if the baseline length reaches about 100 km.

## 1. Introduction

The carrier phase-based Global Positioning System (GPS) has become an essential technique for a wide range of precise positioning applications, such as kinematic positioning and vehicle navigation and guidance. However, there are several constraints on the use of this technique. Firstly, an assurance of satellite signal line-of-sight is a critical requirement, yet GPS signals can be obstructed by buildings, bridges, and even tree foliage. Under these circumstances, the GPS system is unable to continuously carry out its positioning task because of the insufficient number of tracked satellites. Furthermore, the impact of baseline-dependent GPS errors, such as orbit uncertainties, and atmospheric effects, further constrains the applicable baseline length between reference and mobile user receiver to perhaps 10–15 km. These constraints have led to the development of several network-based GPS kinematic positioning techniques, including the virtual reference station approach [1], and the area correction parameter techniques [2]. Additional limitations of using GPS are the relatively low carrier phase data output rate (e.g., typically 1–10 Hz), and the need to deploy more than one GPS antenna to derive full attitude information. Such constraints can be so restrictive that they may hinder the widespread adoption of carrier phases-based GPS techniques for many precise positioning and navigation applications.

Some of the restrictions of carrier phase-based GPS technology can be addressed by its integration with an inertial navigation system (INS). The INS is a self-contained navigation unit providing position, velocity and attitude information based on measurements by its ensemble of sensors (typically a set of accelerometers and gyroscopes). Its disadvantage is that INS navigation accuracy deteriorates rapidly with time due to the presence of sensor biases and a double-integration mechanisation algorithm. However, an appropriate integration of INS with GPS can take advantage of each technology’s strengths, delivering a high data-rate complete navigation solution with both superior short-term and long-term accuracies [3–5]. Even during a GPS signal blockage, it is still possible to carry out positioning in the INS stand-alone mode for short periods of time; the so-called INS bridging mode. The critical duration of the bridging capability varies as a function of the quality of the INS [6]. Nevertheless, the baseline length constraint cannot be overcome using the integrated GPS/INS approach as the quality of double-differenced GPS observations decreases with increasing baseline length. A solution to this problem would be to combine measurements from a number of reference receivers to model the baseline-dependent GPS errors, through applying various parameterisation techniques [7–9]. The use of multiple GPS reference receivers therefore permits the baseline lengths to be much longer than that in the single reference station scenario. As a result, all of the aforementioned constrains of precise kinematic GPS technique would be effectively addressed by augmentation of GPS/INS with multiple reference station carrier phase data.

This paper proposes a GPS/INS integration scheme with multiple GPS references for use in highly precise long-baseline kinematic positioning. This approach utilises measurements from multiple reference stations to model the GPS baseline-dependent errors and to apply them to mobile receiver observations before updating the integration filter. Hence, the applicable baseline length is extended with centimetre-level positioning accuracy. After discussing the concept of the employment of multiple reference stations with the integrated GPS/INS, some technical issues required for the implementation of the algorithm are described. This is followed by the description of measurement simulations and test results with an emphasis on the effects of employing multiple reference stations in the integrated GPS/INS data processing.

## 2. Description of Sensor Integration Concept

**Figure 1.**
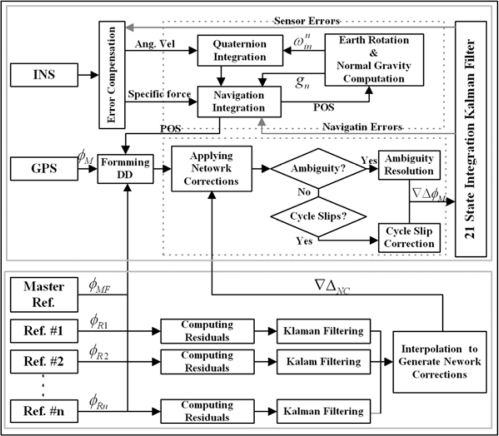
Configuration of the tightly-coupled GPS/INS integration using GPS multiple reference stations.

The proposed integration approach is comprised of two components, namely the stationary reference measurement processing and the mobile positioning instrumentation. The role of the multiple GPS reference stations is to generate the so-called network corrections that model the baseline-dependent errors at a mobile station, whereas the mobile platform component is an integrated GPS/INS device. As illustrated in Figure 1, the mobile component consists of INS mechanisation, GPS carrier phase processing, and the Kalman filter that estimates the INS navigation and sensor errors. Using such an integration scheme, high positioning accuracy can be maintained through the continuous calibration/estimation of the INS errors. The network correction terms substantially reduce the baseline-dependent GPS errors at the mobile station compared with the conventional GPS/INS integration approach based on a single reference station.

## 3. GPS Baseline-Dependent Error Modelling

The GPS baseline-dependent errors are estimated based on the pre-determined coordinates of the reference stations. It is prerequisite to correctly resolve the double differenced (DD) carrier phase ambiguities between the reference stations to generate the accurate corrections. For real-time measurements with baselines over a few tens of kilometres, several integer ambiguity resolution algorithms have been proposed [10–12]. These techniques are characterised by an attempt to form linear combinations with longer wavelengths and less noise, using the L1 and L2 carrier phase measurements. Due to the considerable magnitude of the ionospheric effect in the L1 and L2 carrier phases, the ambiguities of the wide-lane linear combinations are resolved first, and then the L1 ambiguities are fixed from the ionospheric-free measurements. This is followed by computing the L2 ambiguities from those of the resolved L1 and wide-lane combinations. The ambiguity estimation and validation procedure are applied for each step of the resolution. While the least squares ambiguity decorrelation adjustment (LAMBDA) method is used for the ambiguity estimation, the W-ratio test is applied for the validation [13,14].

**Figure 2.**
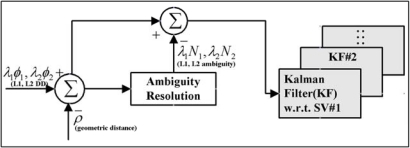
An estimation procedure for GPS baseline-dependent error utilising the Kalman Filter.

The performance of positioning and navigation with the multiple GPS reference stations is largely dependent on the ability of an algorithm to separate the site-dependent errors from the DD residuals computed by subtracting the ambiguities and geometric distances from the DD measurements. Figure 2 illustrates an estimation procedure of the baseline-dependent errors based on a Kalman filtering. The 4-states model has been implemented to describe the dynamics of the baseline-dependent errors (the 1st–3rd state), and the multipath error (the 4th state). As given in Equation (1), a position, velocity, and acceleration (PVA) model is applied for the first three states, whereas the multipath (the 4th state) is modelled as a first-order Markov process [15–16]:

(1) $$\left[\begin{array}{l} {\dot{x}}_{L1}(t)\\ {\dot{x}}_{L2}(t) \end{array}\right]=\left[\begin{array}{ll} F & 0\\ 0 & F \end{array}\right]\left[\begin{array}{l} x_{L1}(t)\\ x_{L2}(t) \end{array}\right]+\left[\begin{array}{l} w_{L1}(t)\\ w_{L2}(t) \end{array}\right]$$

A detailed expression of the dynamic matrix can be given by:

(2) $$F=\left[\begin{array}{llll} 1 & T & \frac{T^{2}}{2} & 0\\ 0 & 1 & T & 0\\ 0 & 0 & 1 & 0\\ 0 & 0 & 0 & e^{-\alpha T} \end{array}\right]$$

where *T* is the sampling interval and *α* is the correction time.

The error states in Equation (1) are as follows:

(3) $$x_{L1}={\left[\begin{array}{llll} \nabla \Delta_{P1} & \nabla \Delta_{V1} & \nabla \Delta_{A1} & \nabla \Delta_{M1} \end{array}\right]}^{T}$$

(4) $$x_{L2}={\left[\begin{array}{llll} \nabla \Delta_{P2} & \nabla \Delta_{V2} & \nabla \Delta_{A2} & \nabla \Delta_{M2} \end{array}\right]}^{T}$$

where ∇Δ*_P_* is the DD baseline-dependent error, ∇Δ*_V_* and ∇Δ*_A_* are the velocity and the acceleration of the error respectively, and ∇Δ*_M_* is the multipath.

## 4. Integrated Mobile GPS/INS Filtering

The so-called corrections, modelled by interpolating the baseline-dependent errors at the reference stations with respect to the position of a mobile station, should be applied for mobile GPS receiver measurements to reduce these errors. Over the past decades, a number of interpolation methods have been proposed, including the linear combination model, distance-based linear interpolation method, and linear interpolation method [17]. However, the performances of these methods are equivalent according to Dai *et al*. [18]. To interpolate the baseline-dependent errors at three or more stations, the coefficients *α* and *β* should be computed using the following equation [9]:

(5) $$\left[\begin{array}{l} \alpha \\ \beta \end{array}\right]={\left(A^{T}A\right)}^{-1}A^{T}C$$

where:

(6) $$A=\left[\begin{array}{cc} \Delta X_{R1} & \Delta Y_{R1}\\ \Delta X_{R2} & \Delta Y_{R2}\\ \vdots & \vdots \\ \Delta X_{R(n-1)} & \Delta Y_{R(n-1)} \end{array}\right]$$

(7) $$C=\left[\begin{array}{c} \nabla \Delta_{P,\text{R1}}\\ \nabla \Delta_{P,\text{R1}}\\ \vdots \\ \nabla \Delta_{P,R(n-1)} \end{array}\right]$$

as well as *ΔX* and *ΔY* are the plane coordinate differences referred to the master reference station (MR), the subscripts *R_i_* indicate the reference station *R_i_*, and the elements of the vector *C* are the baseline-dependent errors.

The mobile platform within the coverage of the reference network can apply the following 2-D linear model to interpolate the baseline-dependent errors:

(8) $$\nabla \Delta_{\mathrm{NC}}=\alpha \Delta X_{\mathrm{MR}}+\beta \Delta Y_{\mathrm{MR}}$$

The integration of GPS with INS has been implemented using a tightly-coupled Kalman filtering technique, which utilises a single filter to process all the data in the DD measurements domain. The integrated processing procedure employed in this study is presented in Figure 1. The error state vector for the filter includes the parameters of the navigation solution and the INS sensor errors. The psi-angle model [e.g., Equations (9–11)] is used to describe the behaviour of INS navigation errors [19].

(9) $${\delta \dot{r}}^{n}=-\Omega_{\mathrm{en}}^{n}{\delta r}^{n}+{\delta \nu }^{n}$$

(10) $${\delta \dot{\nu }}^{n}=-\left(\Omega_{\mathrm{en}}^{n}+2\Omega_{\mathrm{ie}}^{n}\right)\delta \nu^{n}+\delta \nabla^{n}-\Omega_{\psi }f^{n}$$

(11) $${\dot{\psi }}^{n}=-\Omega_{\mathrm{in}}^{n}\psi^{n}+\delta {ɛ}^{n}$$

where *δr*, *δν*, *δψ*, and *δψ* are the position, velocity, and attitude error vector respectively, Ω is the skew-system form of the frame rotation rate vector, δ∇ is the accelerometer error vector, *δε* is the gyro drift vector, and the superscripts and subscripts indicate coordinate frames (e.g., inertial, ECEF, and navigation). The biases and scale factors of the INS sensors are modelled by a random bias and the first-order Gauss-Markov process respectively. Hence, a total of 21 error states are considered in this research. For a more detailed discussion, see, for example, Grejer-Brezinska *et al*. [20], Da [21], and Lee [22].

In this GPS/INS integration, the DD GPS carrier phases are used to update the Kalman filter to estimate the navigation and INS sensor errors. The DD measurements are formed by differencing between two single differences (SD) across two different satellites at each epoch. In the case of a medium baseline up to 100 km, the measurements can be mathematically represented as:

(12) $$\lambda \cdot \Delta \nabla \phi =\Delta \nabla \rho +\underset{\text{the distance}-\text{dependent errors}}{\underset{︸}{\Delta \nabla d\rho -\Delta \nabla di+\Delta \nabla dr}}+\lambda \cdot \Delta \nabla N+\Delta \nabla ɛ$$

where ∇ denotes differencing between two satellites, so that Δ∇ represents a double-differencing, *λ* is the wave length of the carrier phase, *ρ* is the true range or geometric range, *dρ* is the satellite orbit uncertainties, *di* is the ionospheric effect, *dr* is the tropospheric delay, *N* is the integer-ambiguity, and *ε* is the noise.

By applying the network correction, Equation (12) can be re-written as follows:

(13) λ⋅Δ∇ϕ=Δ∇ρ+Δ∇N+Δ∇ɛ

However, Equation (13) contains an integer ambiguity term, which should be correctly resolved to ensure centimetre-level positioning accuracy. Here, an INS-aided ambiguity resolution scheme utilising the INS-predicted position in the float ambiguity estimation has been implemented. This approach enhances the performance of the ambiguity resolution by improving the precision of the DD range estimation. More details on this approach can be found in Lee *et al*. [23]. Equation (13) with the ambiguity resolved is applied for updating the Kalman filter to estimate its error states, which are fed back to correct the inertial solution and sensor measurements. However, it is important to note that the term Δ∇ε in Equation (13) contains not only the noise, but also the un-modelled baseline-dependent errors, which means that the positioning accuracy of the GPS/INS integration is determined by the quality of the corrections modelled by the multiple reference stations.

## 5. Testing and Results with Simulated Measurements

Three sets of simulated GPS and INS measurements have been processed in this section to test the performance of the algorithms implemented and to evaluate the achievable accuracy of the position and attitude parameter estimation.

### 5.1. Measurement Simulation and Processing

All the test measurements were generated using a GNSS/INS simulator consisting of trajectory profile generation, GPS satellite, and INS measurements simulation modules [24]. The original software was modified in the following ways to more realistically model GPS errors: (a) the ionospheric effect is derived from Klobuchar-style coefficients provided by Centre for Orbit Determination in Europe (CODE); (b) the tropospheric delay is computed the Saastamoinen model with the Neil mapping function; (c) the multipath is generated by passing white noise through a first-order Butterworth low-pass filter.

The measurement simulation begins with defining a reference trajectory (the time, coordinate, velocity, and attitude) for a moving vehicle. In the INS data generation, specific force (acceleration) and angular velocity is firstly computed, based on the given trajectory profile. Then, the related sensor errors, accelerometer/gyro bias, scale factor and noise, as well as the effects associated with the Earth’s rotation and gravity, are computed and added to the generated true measurements. All the data generated are stored in a binary format at a rate of 64 Hz. On the other hand, the geometric distances between the receiver and the satellite are initially computed to simulate the GPS observations. The biases, errors and measurement noise are then added to the geometric distance. These simulations were performed with respect to a tactical-grade INS (e.g., gyro drift 5 deg/h and accelerometer bias 500 μg) and dual-frequency geodetic GPS receivers.

**Figure 3.**
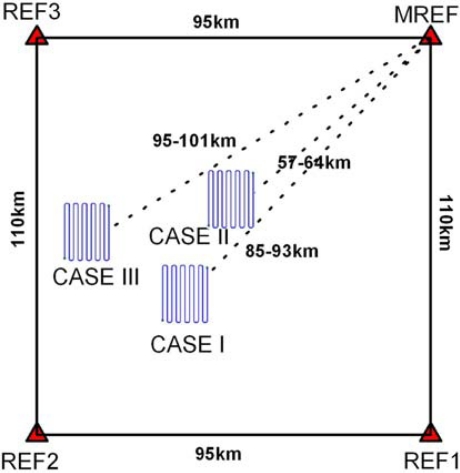
Layout of the GPS multiple reference station and testing areas.

Figure 3 shows a layout of the multiple reference stations and testing area, considered in the simulations. GPS measurements at the four reference stations were generated for a time period of 2,238 seconds, with a separation of 115 km and 95 km in the latitudinal and longitudinal direction respectively.

**Figure 4.**
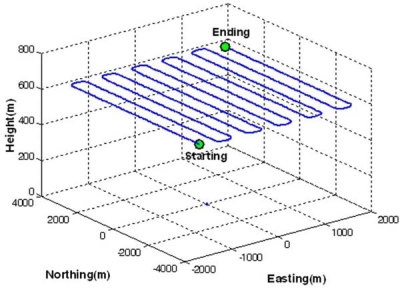
Generated reference trajectory.

Three different scenarios, denoted as CASE1, CASE2, and CASE3, were considered in the GPS/INS simulation, depending on baseline length between the master reference and the mobile station. Because MREF is selected as the master reference station, the baseline lengths range from 57 km to 101 km in these scenarios. Figure 4 depicts an example of the generated reference trajectories (e.g., CASE1), which contains 63 segments defining the vehicle dynamics (e.g., acceleration, deceleration, attitude change). The trajectories were generated with the consideration of an airborne environment, which is a typical example of long-baseline positioning and navigation. The vehicle flew with an average velocity of 55 m/sec (200 km/h) excluding the vehicle turning manoeuvre at the end of flight path. In addition, the flying height above the ground was about 600 m. It was assumed in the trajectory generation that the velocity component along the H-(Up) axis of the navigation frame was zero m/sec unless the pitch and roll angles were changed.

All the simulated measurements were processed in post-mission mode using self-programmed software to estimate the navigation parameters. However, it is important to note that all the algorithms are applicable for the real-time implementation. Although the main goal of this study was to investigate the impact of including the multiple reference stations in the GPS/INS integration in terms of the accuracy of the position and attitude estimation, the performance of the implemented algorithms at each step of data processing was tested, which includes the baseline-dependent errors estimation, the network correction modelling, and the ambiguity resolution

### 5.2. Network Correction Modelling

The data processing of the integrated GPS/INS with multiple reference stations begins with the carrier phase ambiguity resolution between the stations. By applying the procedure described in Section 3, it was possible to fix the ambiguities to their correct values within a few seconds. Because MREF was selected as a master reference station, three baselines, denoted as MREF-REF1, MREF-REF2, and MREF-REF3, were composed in the data processing.

**Figure 5.**
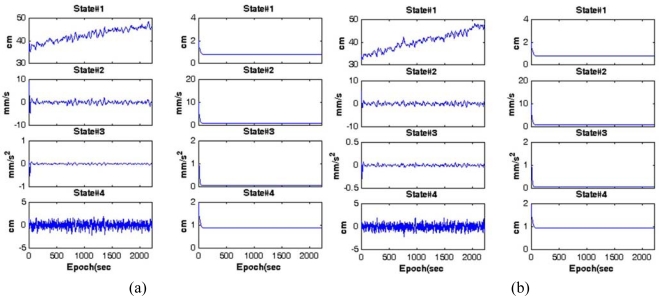
Filtering results to generate the GPS baseline-dependent errors of SV8 (CASE I). **(a)** L1 carrier phases. **(b)** L2 carrier phases.

**Figure 6.**
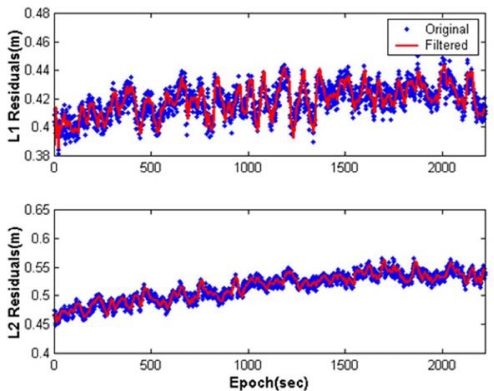
Impact of Kalman filtering in the estimation of the GPS baseline-dependent errors of SV8(CASE I).

The Kalman filters processed the DD residuals computed at the reference stations in parallel to estimate the baseline-dependent errors with the minimising impact of the site-dependent errors. For example, the results from the filtering for the L1 and L2 of space vehicle (SV) 8 in CASE I are shown in Figures 5(a,b). In these figures, the graphs in the first column represent state estimates obtained, whereas those in the second column depict their standard deviations extracted from the diagonal components of the updated covariance matrix. It should be noted from the plots in the second columns that the entire filter states converge with respect to time. To closely observe the impact of the Kalman filtering, the DD residuals of SV8 and those processed by the filter (e.g., the estimated baseline-dependent errors) are illustrated in Figure 6, indicating that the site-dependent errors are reduced.

**Figure 7.**
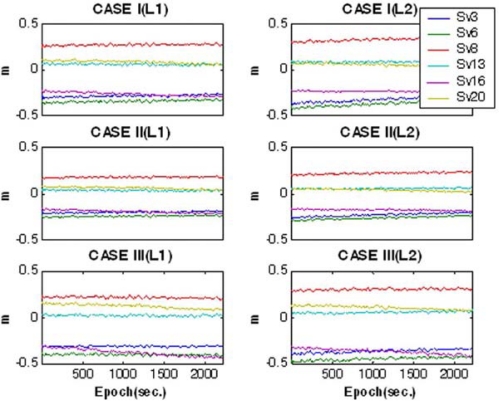
Interpolated GPS baseline-dependent errors (network corrections).

**Figure 8.**
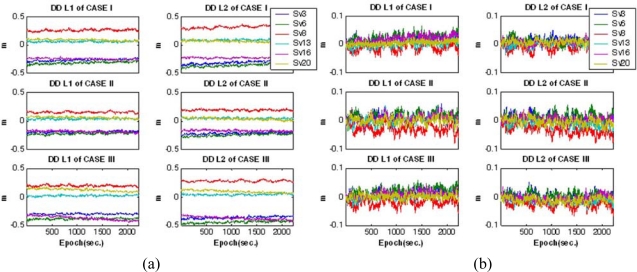
DD residuals of mobile receivers. **(a)** Single-reference station mode. **(b)** Multiple-reference stations mode.

Figure 7 shows the interpolated satellite-by-satellite and epoch-by-epoch L1 and L2 baseline-dependent errors, the so-called network corrections, with respect to the mobile platforms. As illustrated in Figure 8(a), the true DD residuals of the mobile receivers were computed with respect to the reference trajectories to evaluate the performance. Due to the long-baseline between the master reference and the mobile receivers, ranging from 57 km to 101 km, some values mostly representing the baseline-dependent errors may reach a few tens of centimetres, as seen in the figure. To evaluate the accuracy of the modelled network corrections, the DD residuals were re-calculated by applying the network corrections, plotted in Figure 8(b). It can be found from the results that the DD residuals are significantly reduced to a few centimetres in all the cases by applying the network corrections, which represents the achievable modelling accuracy of the corrections in these tests.

### 5.3. Integrated GPS/INS Processing

Ambiguity resolution (AR) is one of the most crucial procedures for achieving the goal of high accuracy carrier phases-based GPS positioning and navigation applications. Due to the existence of the baseline-dependent errors, the ability to correctly resolve the integer ambiguities is restricted to relatively short distances between the reference station and the mobile receiver. To correctly resolve the ambiguities, the residual errors of the DD carrier phases should be theoretically smaller than a half-wavelength, corresponding to about 10 cm and 12 cm for the L1 and L2 phase observations, respectively. It is almost impossible to resolve the correct ambiguities using the single reference AR technique in the case of the medium baseline. However, the proposed integration technique is expected to correctly fix the ambiguities by modelling the baseline-dependent errors based on the multiple reference stations and using the INS-predicted position obtained from its mechanisation. An instantaneous AR procedure with single-epoch observations was carried out to demonstrate its performance. The results are summarised in Table 1 in terms of the success rate of the ambiguity resolutions. As expected, the table shows that the instantaneous AR becomes possible with the proposed approach.

**Figure 9.**
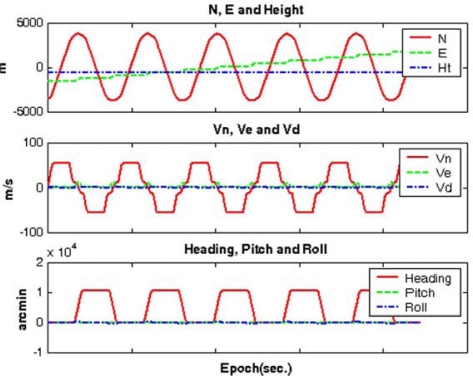
Example of the estimated navigation parameters (CASE I).

**Figure 10.**
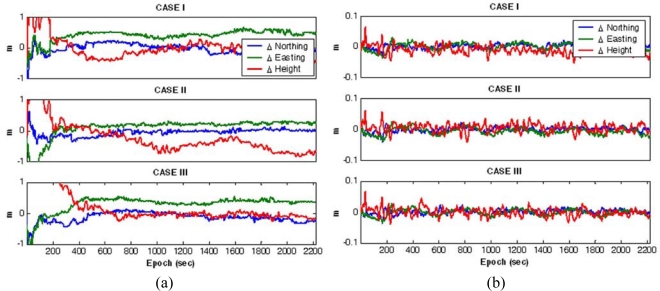
Coordinate differences between the references and estimates. **(a)** Single-reference station mode. **(b)** Multiple-reference stations mode.

Figure 9 shows the navigation parameters estimated by the Kalman filter during the vehicle manoeuvre. To demonstrate the benefit of including multiple reference stations, the simulated measurement sets were processed twice with different modes: the single-reference station mode and the multiple-references stations mode. A comparison between the reference trajectories and estimated coordinates was used to determine the achievable accuracy. Figure 10(a) shows the coordinate differences for the GPS/INS with the single-reference scenario, whereas Figure 10(b) depicts those of the GPS/INS with the multiple-reference stations. In addition, Table 2 statistically summarises the comparison. It can be observed from the results that the accuracy for the GPS/INS with the single-reference reaches a few decimetres, mostly reflecting the baseline-dependent errors in the observations. On the other hand, these results reveal that centimetre-level positioning would be possible with the proposed integration scheme even if the baseline length is about 100 km. Furthermore, although the baseline lengths considered in study are different by a few tens of kilometres, the achievable positioning accuracies are similar. This is due to the fact that, as evaluated in Figure 8(b), the baseline-dependent errors are modelled with equivalent accuracies.

Table 3 summarises a comparison of the attitude parameters between the reference and estimate values. Comparing the results from the single-reference mode with those from the multiple-references mode reveals that marginal improvement is only found in the mean values. These results are attributed to the fact that the attitude accuracy of an INS does not depend much on the quality of the GPS measurements used in the filter update, but is largely determined by gyro drift rates.

## 6. Concluding Remarks

An integration of GPS/INS with multiple-reference stations for long-baseline kinematic positioning has been proposed in this paper, with the objective of improving the accuracy of the position estimation in the case of baseline lengths up to about 100 km. The algorithms concerned with implementing the proposed scheme are addressed, which include the AR between reference stations, the estimation of the baseline-dependent errors, the network correction modelling, and the integrated GPS/INS processing with an emphasis on the Kalman filter design. Three GPS and INS simulated measurement sets on mobile platforms were processed to characterise not only the performance of the algorithms implemented, but also the achievable accuracy of the position and attitude estimation. The results show that the accuracy of the position component was significantly improved to be a few centimetres through modelling the baseline-dependent errors based on the multiple reference stations. Because the accuracy of the attitude estimation based on INS depends primarily on the quality of the gyro sensors, marginal improvement was observed in the component. In conclusion, more research on quality control algorithms for the integration and further tests with real data sets will be carried out in the near future.

## Figures and Tables

**Table 1.** Success rate of the ambiguity resolutions (epoch-by-epoch).

**CASE**	**I**	**2**	**3**

**Success Rate**	99.7%	100.0%	99.0%

**Table 2.** Statistical summary of the coordinate differences between the references and estimates (unit: centimetres).

**CASE**	**Single-reference GPS/INS**						**Multiple-references GPS/INS**					
	**Δ Northing**		**Δ Easting**		**Δ Height**		**Δ Northing**		**Δ Easting**		**Δ Height**	
	**Mean**	**Std.**	**Mean**	**Std.**	**Mean**	**Std.**	**Mean**	**Std.**	**Mean**	**Std.**	**Mean**	**Std.**
I	3.8	±.16.5	41.5	±.17.1	41.7	±23.8	0.3	±0.7	0.1	±1.1	1.1	±1.3
II	5.1	±11.4	10.5	±32.8	11.7	±34.7	0.0	±0.7	0.5	±1.0	0.6	±1.3
III	13.4	±17.5	29.8	±26.3	32.7	±31.6	0.1	±0.7	0.1	±1.0	0.1	±1.3

**Table 3.** Statistical summary of the attitude parameter differences between the references and estimates (unit: arc-minutes).

**CASES**	**Single-reference GPS/INS**						**Multiple-references GPS/INS**					
	**Δ Heading**		**Δ Pitch**		**Δ Roll**		**Δ Heading**		**Δ Pitch**		**Δ Roll**	
	**Mean**	**Std.**	**Mean**	**Std.**	**Mean**	**Std.**	**Mean**	**Std.**	**Mean**	**Std.**	**Mean**	**Std.**
I	2.50	±2.50	0.69	±0.72	2.69	±1.32	1.92	±2.26	0.53	±0.64	2.67	±1.27
II	2.11	±2.61	0.72	±0.77	2.56	±1.33	1.88	±2.28	0.53	±0.64	2.66	±1.27
III	2.62	±2.71	0.74	±0.77	2.69	±1.32	1.93	±2.28	0.53	±0.64	2.68	±1.27
